# Metabolic engineering strategies to produce medium-chain oleochemicals via acyl-ACP:CoA transacylase activity

**DOI:** 10.1038/s41467-022-29218-3

**Published:** 2022-03-25

**Authors:** Qiang Yan, William T. Cordell, Michael A. Jindra, Dylan K. Courtney, Madeline K. Kuckuk, Xuanqi Chen, Brian F. Pfleger

**Affiliations:** 1 Department of Chemical and Biological Engineering, University of Wisconsin-Madison, Madison, WI 53706 USA; 2 DOE Center for Advanced Bioenergy and Bioproducts Innovation, University of Wisconsin-Madison, Madison, WI 53706 USA; 3 Microbiology Doctoral Training Program, University of Wisconsin-Madison, Madison, WI 53706 USA

**Keywords:** Metabolic engineering, Metabolic engineering, Applied microbiology

## Abstract

Microbial lipid metabolism is an attractive route for producing oleochemicals. The predominant strategy centers on heterologous thioesterases to synthesize desired chain-length fatty acids. To convert acids to oleochemicals (e.g., fatty alcohols, ketones), the narrowed fatty acid pool needs to be reactivated as coenzyme A thioesters at cost of one ATP per reactivation - an expense that could be saved if the acyl-chain was directly transferred from ACP- to CoA-thioester. Here, we demonstrate such an alternative acyl-transferase strategy by heterologous expression of PhaG, an enzyme first identified in *Pseudomonads*, that transfers 3-hydroxy acyl-chains between acyl-carrier protein and coenzyme A thioester forms for creating polyhydroxyalkanoate monomers. We use it to create a pool of acyl-CoA’s that can be redirected to oleochemical products. Through bioprospecting, mutagenesis, and metabolic engineering, we develop three strains of *Escherichia coli* capable of producing over 1 g/L of medium-chain free fatty acids, fatty alcohols, and methyl ketones.

## Abstract (web summary)

Microbial production of oleochemicals involves strategies of expressing thioesterase to narrow the substrate pool for the termination enzyme at the expense of one ATP. Here, the authors developed an alternative energy-efficient strategy to use of an acyl-ACP transacylase to produce medium chain oleochemicals in *E. coli*.

## Introduction

Oleochemicals are a class of aliphatic hydrocarbons that are industrially derived from petroleum, animal fats, or oilseed crops. Depending on the terminal reductive state of the acyl chain, oleochemicals can be classified as fatty acids, alcohols, aldehydes, ketones, esters, olefins, alkanes, etc. Oleochemicals are also categorized by size (i.e., acyl-chain length). Medium-chain oleochemicals are defined as compounds with an acyl moiety of 8–12 carbons and are of particular interest due to their applications as a commodity and specialty chemicals^1,2^. For example, medium-chain fatty acids are used as herbicides, antimicrobials, and precursors for lubricant synthesis^3^. Medium-chain methyl ketones are used as flavors, floral fragrances, insecticides, and renewable liquid transportation fuels^4,5^. Medium-chain alcohols are used as surfactants, as additives in biodiesel, cosmetics, and consumer products. The unique molecular and chemical properties of medium-chain oleochemicals and their limited natural abundance make them attractive targets for biosynthesis.

The final enzymatic reactions in oleochemical biosynthesis use acyl-thioesters as substrates, making high-flux to desired chain length acyl-thioesters a key element to any metabolic engineering strategy. Thus far, two approaches have been applied to produce medium-chain acyl-thioesters in microbes: thiolase-driven β-reduction cycles (i.e., reversal of β-oxidation)^6^ and thioesterase-driven diversion of fatty acid biosynthesis intermediates^2,7^. The reverse β-oxidation pathway incorporates a reversible, thiolase-catalyzed Claisen condensation (e.g., FadA) with acetyl-CoA as the donor to bypass the energy consumption required for producing malonyl-CoA in fatty acid biosynthesis. The 1-ATP savings per elongation provides reversal of β-oxidation with the highest theoretical yield of all oleochemical biosynthesis routes at the cost of losing the strong driving force provided by the decarboxylative Claisen condensation in fatty acid biosynthesis. β-reduction was used to demonstrate the production of both medium-chain fatty acids and fatty alcohols^6,8–11^ in high yields, albeit with poor selectivity to specific chain length products. This is in part due to the competition between termination reactions (cleavage or reduction, respectively) and thiolase-catalyzed extension of acyl-CoA thioesters. To date, strategies to bias termination at a particular chain length have yet to be demonstrated and better results have come from finding ways to accumulate pools of desired chain-length acyl-CoAs made via fatty acid biosynthesis to avoid the competition entirely.

Plants synthesize novel oils containing medium-chain acyl groups by expressing selective acyl-ACP thioesterases in the chloroplast to generate free fatty acids of the desired size^7^. Once made, the free fatty acids are transported to the cytosol for reactivation as acyl-CoA and subsequent incorporation into storage lipids^12^. In microbes, plant acyl-ACP thioesterases can be leveraged to produce free fatty acids of desired chain length. Additional genetic modifications are needed to enable the conversion of the free fatty acids to the desired oleochemical form (e.g., alcohol, ketone, ester). Unlike plants where biochemistries can be compartmentalized, bacteria express enzymes for both fatty acid biosynthesis and catabolism in the same locations. In order to accumulate a pool of desired acyl-CoA’s β-oxidation must be blocked by eliminating all enzymes that catalyze one of the four reactions in the cycle. Depending on the desired product form, different β-oxidation steps are targeted (e.g., acyl-CoA dehydrogenase, FadE, for fatty alcohol production; thiolase, FadA/FadI, for methyl ketone production) to produce the substrate for termination enzymes (e.g., saturated acyl-CoA for acyl-CoA reductase conversion to fatty alcohol; β-ketoacyl-CoA for β-ketothioesterase conversion to methyl ketone). The last steps in the metabolic engineering strategy are an expression of a specialized acyl-ACP thioesterase (to produce desired free fatty acids), expression of acyl-CoA synthetase (to activate the free fatty acid), and expression of the desired termination enzyme. Highly active acyl-ACP thioesterases have been identified from natural sources and others have been engineered or evolved in the laboratory to produce octanoic acid, decanoic acid, dodecanoic acid, and tetradecanoic acid^13–17^. For instance, a highly active variant of a *Cuphea palustris* thioesterase FatB (referred to ^*Cp*^FatB*) was identified using a ^*Cp*^FatB random mutagenesis library and a growth selection based on the lipoic acid requirement of *Escherichia coli*. *E. coli* strain NHL17 (MG1655 *ΔaraBAD ΔfadD::P*_*trc-*_^*Cp*^fatB*) produced 1.7 g/L octanoic acid with >90% specificity from 20 g/L glycerol^14^. The highly active C8-specific ^*Cp*^FatB* enzyme was utilized to produce 1-octanol by expressing an acyl-CoA synthetase (^*Mt*^FadD6) from *Mycobacterium tuberculosis* to reactivate octanoic acid at the cost of 1 mole ATP and expressing an acyl-CoA reductase from *Marinobacter aquaeolei* (^*Ma*^ACR) to convert octanoyl-CoA to 1-octanol. The resulting *E. coli* strain NHL24 produced 1.3 g/L 1-octanol^18^. In a separate study, 2-heptanone was produced by converting octanoyl-CoA to β-ketooctanoyl-CoA via an acyl-CoA oxidase from *Micrococcus luteus* (Mlut_11700) and an endogenous bi-functional dehydrogenase ^*Ec*^FadB. Subsequently, β-ketooctanoyl-CoA was hydrolyzed by an β-ketoacyl-CoA thioesterase ^*Ps*^FadM from *Providencia sneebia* (^*Ps*^FadM) and the resulting β-ketooctanoic acid was decarboxylated non-enzymatically to yield 2-heptanone. Strain *E. coli* TRS12 (MG1655 Δ*araBAD* Δ*fadD::P*_*trc*_-^*Cp*^fatB1* Δ*fadA* Δ*fadE* Δ*fadI* Δ*fadR*) harboring pTRC99a-^*Mt*^*fadD6*-^*Ps*^*fadM* and pACYC-^*Ml*^*mlut_11700* plasmids produced up to 4 g/L 2-heptanone in fed-batch bioreactor experiments^4^. Although successful, this thioesterase strategy could be further improved by replacing the futile cycle of thioester cleavage and formation with a direct acyl transfer.

In nature, some bacteria such as *Pseudomonads*, accumulate polyhydroxyalkanoate (PHA) as a means of storing carbon and energy. PHA polymerization requires a supply of (*R*)-3-hydroxyacyl-CoAs (PHA monomers), which can be derived from either fatty acid biosynthesis or β-oxidation. In 1998, PhaG, an enzyme found in *Pseudomonas putida* and *Pseudomonas aeruginosa*, was identified as the enzymatic link between fatty-acid biosynthesis and PHA biosynthesis^19,20^. PhaG was hypothesized to catalyze the transfer of the (*R*)-3-hydroxyacyl moiety from the ACP thioester to CoA. In vitro experimental results showed a time course of CoA release by incubating purified PhaG, (*R*)-3-hydroxydecanoyl-CoA and holo-ACP, indicating PhaG catalyzes a reversible transacylase reaction^21^. In subsequent studies, researchers overexpressed PhaG and observed increased PHA content in cells and an increased fraction of medium-chain length (mcl) 3-hydroxyalkanoate units in the polymer^22–26^. Given these results, we hypothesized that PhaG could provide a similar role in linking fatty acid biosynthesis with the creation of tailored pools of acyl-CoAs and oleochemical products.

**Fig. 1:**
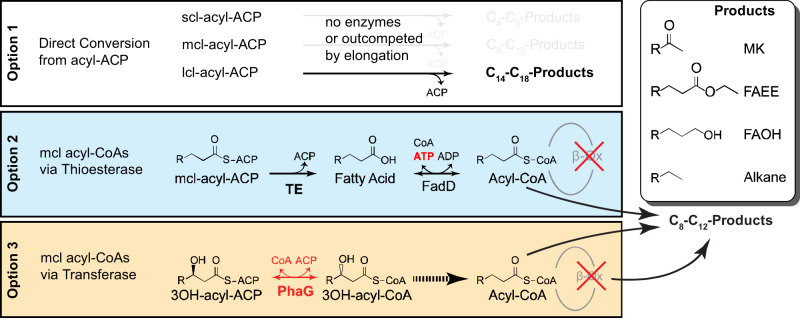
Metabolic pathways for producing medium-chain oleochemicals via fatty acid biosynthesis. Oleochemical products such as methyl ketones (MK), fatty acid ethyl esters (FAEE), fatty alcohols (FAOH), and alkanes are made by enzymes that act on acyl-thioesters. Many oleochemical-producing enzymes can act on long-chain acyl-ACPs (Option 1) but fail to produce medium or short-chain products in high amounts without additional genetic modifications. The reduced activity is likely caused by lower concentrations of shorter chain length acyl-ACPs and/or reduced specific activity on these substrates, particularly when compared to enzymes in fatty acid biosynthesis that elongate the same substrates. Alternatively, if β-oxidation is blocked (often by deleting enzymes catalyzing the appropriate step in the cycle), higher concentrations of mcl-acyl-CoA’s can be provided to saturate oleochemical-producing enzymes. This acyl-CoA pool can be created either by the combination of thioesterase (TE) and acyl-CoA synthetase (e.g., FadD) activity (Option 2) or by acyl-transferase activity (Option 3). Transferase activity (e.g., PhaG) saves one ATP per product relative to Option 2. Detailed pathways for Options 2 and 3 are illustrated in Supplementary Fig. 1.

In the present study, we demonstrate a PhaG-dependent pathway as an alternative strategy to link fatty acid biosynthesis and oleochemical production. We validate the ability of *P. putida* PhaG to direct flux towards oleochemical synthesis at rates comparable to thioestereases. Using computational bioprospecting tools, we identify seven homologs of ^*Ppu*^PhaG and evaluate their in vivo activities. The PhaG variant from *Pseudomonas koreensis* produces 1.6-fold more methyl ketones than the ^*Ppu*^PhaG variant. We construct a random mutagenesis library of ^*Pk*^PhaG and isolate seventeen beneficial mutations that increase octanoic acid production 3.3–16.3-fold above strains expressing the original ^*Pk*^PhaG. We use these improved enzymes to construct strains capable of producing three demonstration oleochemicals—free fatty acids, fatty alcohols, and methyl ketones. Strains expressing the PhaG-dependent pathway are capable of producing 1.1 g/L C_8_–C_14_ free fatty acids, 1.5 g/L C_7_–C_15_ methyl ketones, and 1.1 g/L C_8_–C_16_ fatty alcohols depending on the tailoring enzymes co-expressed. These titers demonstrate that PhaG is a useful alternative for medium chain length oleochemical synthesis and a promising target for future protein engineering to guide substrate selectivity. Yields remain ~50% of the theoretical limit, on par with demonstrations of many thioesterase-utilizing strategies. Continued improvement of PhaG-driven pathways will allow strains to access higher theoretical yields than current thioesterase strategies (Fig. 1, Supplementary Table 1 and Supplementary Method 1).

## Results and discussion

### Genetic studies support PhaG has acyl-ACP:CoA transacylase activity

Homologs of ^*Ppu*^PhaG have been used extensively as a means of enhancing the production of mcl PHA in bacteria^19,27–31^. That said, the specific activities catalyzed by ^*Ppu*^PhaG are debated. In vitro studies have confirmed the ability of PhaG to generate 3-hydroxyacyl-ACP from the corresponding CoA species and holo-ACP^20,21^. This is the reverse reaction of the one desired for oleochemical production studies and no in vitro data on acyl-ACPs, the substrate in the forward direction is available. From 1998 to 2012, PhaG was generically called a 3-hydroxyacyl ACP:CoA transacylase, based on in vitro data. In 2012, Nomura and co-workers challenged the name and the ability of the enzyme to perform the transferase reaction^30^. In this study, *E. coli* BL21 cells harboring a plasmid for expressing an mcl-PHA polymerase (PhaC1) were transformed with plasmids expressing PhaG and/or a CoA ligase from *P. putida*. Cells expressing both enzymes produced more than ten times the amount of PHA than those lacking the CoA ligase. The conclusion drawn from this study is that the ligase is needed for high-flux PHA generation and PhaG acts primarily as a thioesterase. Subsequent papers have used the name thioesterase, but have not provided further evidence to support the presence of thioesterase activity. In contrast, prior studies demonstrated that co-expression of TesB, a promiscuous CoA thioesterase enhanced the production of 3-hydroxy-fatty acids in both *E. coli* and *P. putida*^32,33^. TesB was similarly used to produce 3-hydroxy fatty acids as precursors to methyl esters^34^.

**Fig. 2:**
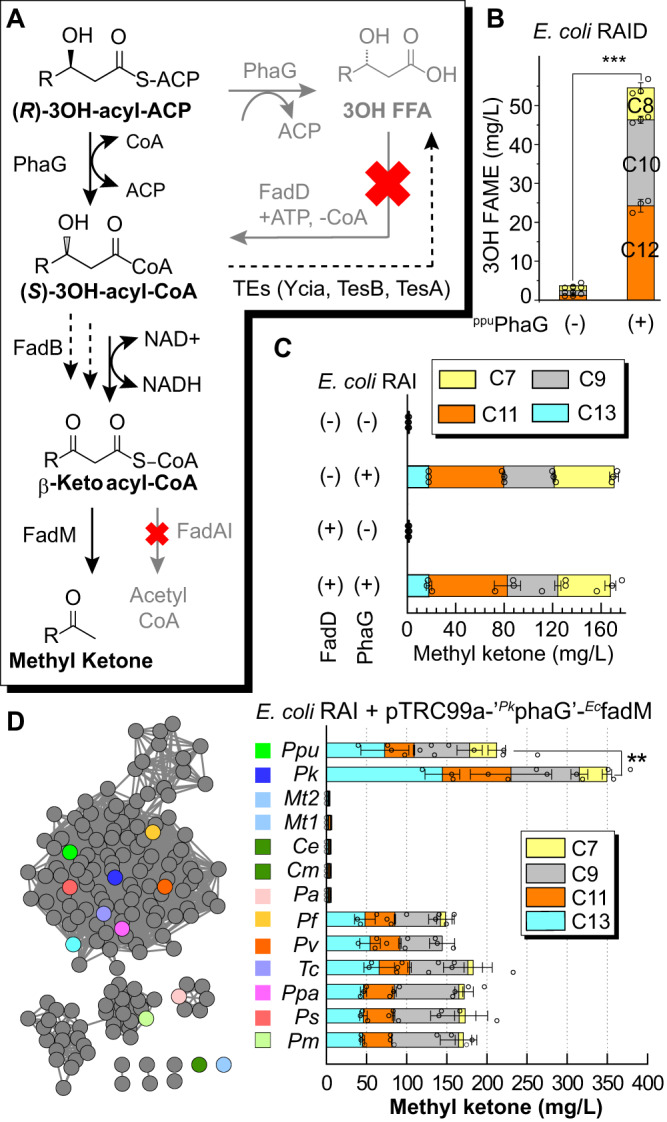
PhaG transacylase activity in vivo. **A** Metabolic pathways used to test for the presence of PhaG-dependent thioesterase and/or transferase activity. **B** In cells lacking FadR, FadA, FadI, and FadD (*E. coli* RAID harboring pTRC99a-^*Ppu*^*phaG*), PhaG expression leads to the production of 50 mg/L medium-chain 3-hydroxy fatty acids (*n* = 3 biologically independent samples). These products could be generated by either direct PhaG thioesterase activity on medium-chain acyl-ACPs or by CoA thioesterase activity on stranded pools of 3-hydoxyacyl-CoAs made via PhaG transferase activity. ***P = 0.0005 was analyzed based on student two-tailed *t* test assuming unequal variances. **C** Co-expression of PhaG, and FadM in *E. coli* RAI (MG1655 *ΔfadR ΔfadA ΔfadI* harboring pTRC99a-^*Ppu*^*phaG*-^*Ec*^*fadM*) results in the production of 150 mg/L of medium-chain methyl ketones (*n* = 3 biologically independent samples). FadD expression did not impact methyl ketone production indicating that free fatty acid activation is not required for PhaG-dependent methyl ketone production. **D** A two-dimensional cluster map created with the Enzyme Similarity Tool^37^ displays the sequence similarity of PhaG variants tested in bioprospecting studies. Quantitative pairwise percent amino-acid identity of each homolog can be found in Supplementary Figure 2. Colored boxes and dots are used to indicate the sequences tested. Mean methyl ketone titers for constructs of PhaG homologs using *E. coli* RAI harbor pTRC99a-‘^*Ppu*^*phaG*’-^*Ec*^*fadM* (*n* = 3 biologically independent samples). All cultures were grown in Clomburg medium containing 20 g/L glycerol at 30 °C and shaking at 250 r.p.m. **P = 0.009 was analyzed based on student two-tailed *t* test assuming unequal variances. All data represent the mean ± s.d. of biological triplicates. Source data underlying B–D are provided as a Source Data file.

The complicated history motivated us to confirm that PhaG had substantial transferase activity. Therefore, we compared the metabolic product profiles (looking for production of methyl ketones or 3-hydroxy fatty acids) of specifically engineered strains of *E. coli* (MG1655 Δ*fadA*, Δ*fadI*, Δ*fadD*, Δ*fadR*, pTRC99a-^*Ppu*^*phaG*-^*Ec*^*fadM*) to determine if heterologously expressed PhaG demonstrated more thioesterase or transacylase activity. Strains were designed to create a 3-hydroxy fatty acid product sink to indicate potential PhaG thioesterase activity and a methyl ketone product sink for PhaG transacylase activity (Fig. 2A). Deletion of *fadD* removes the dominant acyl-CoA synthetase activity and prevents the reactivation of free fatty acids generated by either FadM or PhaG. Deletion of *fadA* and *fadI* removes known thiolase activities from *E. coli* and blocks β-oxidation from catabolizing any acyl-CoAs produced in vivo. Deletion of *fadR* removes repression of *fadB* expression and thereby upregulates a bi-functional enoyl-CoA hydratase/3-hydroxyacyl-CoA dehydrogenase responsible for converting 3-hydroxyacyl-CoA thioesters to 3-ketoacyl-CoA thioesters. FadM^4,35^ is overexpressed to provide 3-ketoacyl-CoA thioesterase activity, resulting in the conversion of any 3-hydroxyacyl-CoAs generated by PhaG to the corresponding methyl ketones. Cultures of *E. coli* RADI strain harboring pTRC99a-^*Ppu*^*phaG*-^*Ec*^*fadM* were grown in Clomburg media at 30 °C for 48 h. Culture samples were extracted and derivatized for GC/FID and GC/MS analysis. The samples contained a total of 170 mg/L C_7_–C_13_ methyl ketones but no detectable 3-hydroxy methyl esters (Fig. 2B) indicating that ^*Ppu*^PhaG functions primarily as a 3-hydroxyacyl ACP:CoA transacylase. Strains expressing FadD (*E. coli* RAI strain pTRC99a-^*Ppu*^*phaG*-^*Ec*^*fadM*) produced equivalent amounts of methyl ketones, indicating that the carbon flux for methyl ketone synthesis is not enhanced by FadD-catalyzed free fatty acid reactivation. Strains lacking PhaG overexpression (*E. coli* RADI strain pTRC99a-^*Ec*^*fadM*) produced small amounts (<1 mg/L) of methyl ketones (Fig. 2B) that have been previously observed in strains expressing FadM^4,35^. Strains lacking ^*Ec*^FadM overexpression (*E. coli* RADI strain pTRC99a-^*Ppu*^*phaG*) contained a total of 55 mg/L C_8_–C_12_ 3-hydroxy methyl esters (Fig. 2C) consistent with prior studies^32,33^. Together, these data suggest that 3-hydroxy fatty acid production observed in past studies likely comes from thioesterase activities encoded by native enzymes (e.g., YciA, FadM, TesB can potentially catalyze the cleavage of (*R*)-3-hydroxyacyl-CoA to 3-hydroxy fatty acids) that are outcompeted by the methyl ketone synthesis pathway we introduced.

### Bioprospecting identifies active PhaG variants

Next, we sought to identify higher activity variants through bioprospecting. We conducted a homology search based on the *Pseudomonas putida* KT2440 PhaG (^*Ppu*^PhaG) sequence using the Basic Local Alignment Search Tool (BLAST)^36^ to identify candidate protein sequences. BLAST hits were sorted using the Enzyme Similarity Tool^37^. Among the homologous sequences that have similarity >45%, we found >95% of sequences belong to *Pseudomonas* species, indicating that PhaG provides activity unique from other PHA-producing bacteria^38^. We selected 13 PhaG homologs, which had a protein sequence similarity range of 24–88% based on a pairwise comparison, shown in Fig. 2D and Supplementary Figure 2. We were particularly interested in homologs from *Mycobacteria* and *Corynebacteria* species because of their potential to interface with substrates linked to the ACP domain of type I fatty acid synthase (FAS) found in these species^39^. The activity of PhaG homologs was assayed in vivo by monitoring methyl ketone production using *E. coli* RADI harboring a pTRC99a-’^*Pk*^*phaG*’-^*Ec*^*fadM* plasmid. Most PhaG variants generated similar methyl ketone titers to ^*Ppu*^PhaG albeit with reduced levels of 2-heptanone. The PhaG variants from *Mycobacteria* and *Corynebacteria* failed to produce methyl ketones. We found that the *P. koreensis* ^*Pk*^PhaG showed the highest production at 350 mg/L total methyl ketone, 1.6 times higher than the ^*Ppu*^PhaG, shown in Fig. 2D. The methyl ketone profile included 34 mg/L 2-heptanone, 85 mg/L 2-nonanone, 85 mg/L 2-undecanone, and 145 mg/L 2-tridecanone indicating a broad activity against medium-chain 3-hydroxyacyl-ACPs. The product distribution did not vary significantly across the tested variants. Therefore, substrate preference will need to be addressed with protein engineering efforts analogous to those targeted to acyl-ACP thioesterases^15^.

### Metabolic engineering to enhance methyl ketone production

**Fig. 3:**
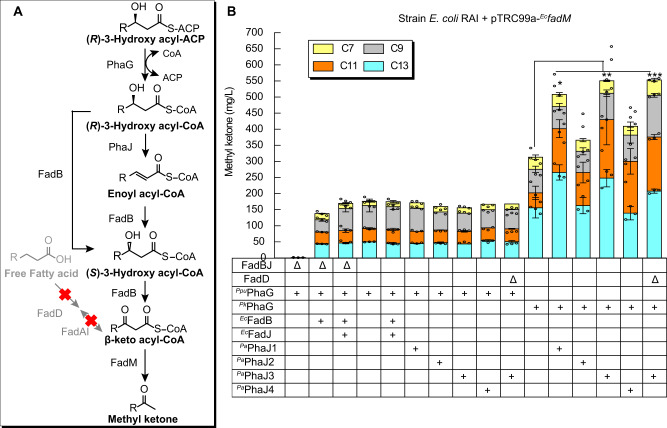
Metabolic engineering to enhance methyl ketone production. **A** Detailed metabolic pathway is used to enable PhaG-dependent production of methyl ketones. **B** Evaluation of alternative routes of isomerizing 3-hydroxyacyl-CoAs with respect to PhaG activity. A series of mutations were made to the base strain, *E. coli* RAI (MG1655, *ΔaraBAD ΔfadR ΔfadA ΔfadI*) (*n* = 3 biologically independent samples). Cultures of each strain harboring pTRC99a-^*Ppu*^*phaG*-^*Ec*^*fadM* or pTRC99a-^*Pk*^*phaG*-^*Ec*^*fadM* were grown for 48 hrs in Clomburg medium containing 20 g/L glycerol at 30 °C and shaking at 250 r.p.m. All data represent the mean ± s.d. of biological triplicates. ***P = 0.0001, **P = 0.003, *P = 0.02 were analyzed based on student two-tailed *t* test assuming unequal variances. Source data underlying B are provided as a Source Data file.

A central tenet of metabolic engineering states that enzyme activity must be balanced across a metabolic pathway to minimize unwanted accumulation of intermediates and maximize pathway flux. In order to assess the relative activity of PhaG to pathway flux, we varied co-overexpression of ^*Ppu*^PhaG, the more-active ^*Pk*^PhaG, and enzymes that convert 3-hydroxyacyl-CoAs to 3-ketoacyl-CoAs. In particular, we were concerned about the relative activity of ketoreductases on the two 3-hydroxyacyl-CoA stereoisomers. PhaG generates (*R*)-3-hydroxyacyl-CoA for direct polymerization into PHA. In contrast, β-oxidation passes through (*S*)-3-hydroxyacyl-CoAs. FadB, the dual-function enoyl-CoA hydratase, and dehydrogenase, can isomerize (*R*)-3-hydroxyacyl-CoA via the corresponding enoyl-acyl-CoA, likely at a reduced rate relative to its regular substrate isomer^40–43^. PHA-producing bacteria solve this problem by expressing an alternative enoyl-CoA hydratase (PhaJ) that can generate the preferred *R*-isomer (Fig. 3A) from β-oxidation intermediates. We selected ^*Ec*^FadB and ^*Ec*^FadJ, an anaerobically expressed FadB homolog, and four previously studied PhaJ variants from *P. aeruginosa*^44^. These enzymes were important optimization points because deletion of *fadB* and *fadJ* eliminated PhaG-dependent methyl ketone production (Fig. 3B).

A combination of ^*Ec*^FadB, ^*Ec*^FadB/^*Ec*^FadJ, and four ^*Ppu*^PhaJ homologs was cloned into an operon linked to a P_*TRC*_ promoter on a pACYC vector. Each of these vectors was co-expressed with pTRC99a-^*Ppu*^*phaG*-^*Ec*^*fadM* or pTRC99a-^*Pk*^*phaG*-^*Ec*^*fadM* in *E. coli* RAI. Cultures of each strain were grown at 30 °C for 48 h. Methyl ketones were extracted from culture samples and quantified by GC/FID (Fig. 3). Strains expressing ^*Ppu*^PhaG all produced ~160 mg/L of methyl ketones with similar distributions to prior experiments. In contrast, when ^*Pk*^PhaG was expressed, methyl ketone titers increased two- threefold relative to the corresponding ^*Ppu*^PhaG strains. In this series, co-expression of PhaJ1 and PhaJ3 had the biggest impact on methyl ketone titer, surpassing 0.5 g/L in cultures of the best strains. These experiments indicate that 3-hydroxyacyl-CoA isomerization can be a limiting step when PhaG transacylase activity is increased. Further, these experiments suggested that titers could be improved with more-active PhaG enzymes.

### Random mutagenesis improves PhaG activity

**Fig. 4:**
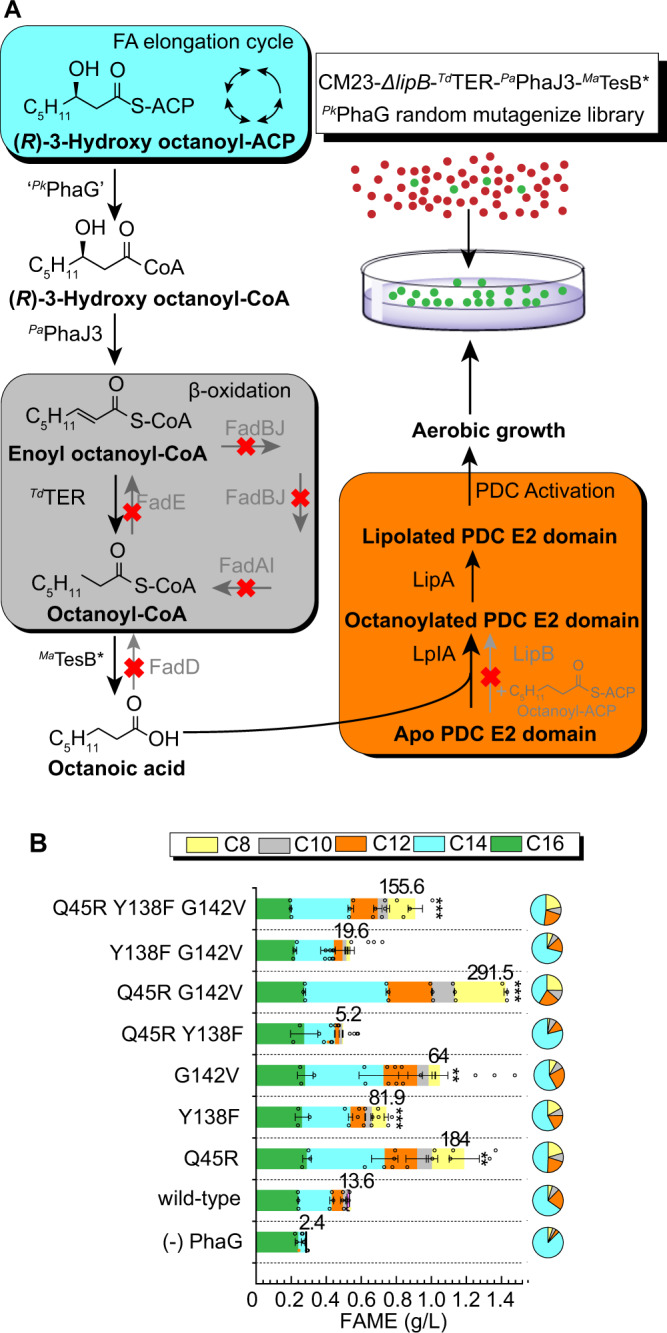
Protein engineering enhances PhaG activity. **A** Metabolic pathways involved in PhaG complementation of a Δ*lipB* driven lipoic acid auxotrophy in *E. coli* CM23 *ΔlipB*. ^*Ec*^FadD deletion blocks the reactivation of fatty acids. ^*Ec*^FadBJ deletion blocks hydration and dehydrogenation of octenoyl-CoA to β-ketooctanoyl-CoA. ^*Ec*^FadAI deletion blocks thiolase-driven elongation or reduction of acyl-CoA chains. LipB deletion blocks activation of apo pyruvate dehydrogenase complex (PDC) E2 domain to octanoylated PDC E2 domain. (*R*)-3-hydroxyoctanoyl-CoA is dehydrated and hydrogenated by ^*Pa*^PhaJ3 and ^*Td*^TER to generate octanoyl-CoA. Octanoyl-CoA is hydrolyzed by ^*Ma*^TesB* to release octanoic acid and CoA. Octanoic acid is ligated to Apo PDC E2 domain by LplA. LipA creates the functional lipoylated E2 domain restoring PDH activity. A functional selection was performed by introducing PhaG variants into the selection strain and plating cells on minimal MOPS-glucose agar. In the selection strain, ^*Ma*^TesB* and ^*Td*^TER were expressed from pTRC99a, ^*Pa*^PhaJ3 was expressed from pACYC, ^*Pk*^PhaG, and other variants, were expressed from a low-copy vector, pBTRCK. **B** FAME analysis of cultures harboring PhaG variants containing combinations of point mutations identified in selection experiments (*n* = 3 biologically independent samples). *E. coli* CM23 harboring pBTRCK-’^*Pk*^*phaG*’ + pACYC-^*Pa*^*phaJ3* + pTRC99a-^*Td*^*TER*-^*Ma*^tesB* were cultured in test tubes containing 5 mL Clomburg 20 g/L glycerol and 1 mM IPTG at 30 °C for 48 hrs. All data represent the mean ± s.d. of biological triplicates. ***P = 0.0001 (Q45R Y138F G142V vs. wild-type), ***P = 0.0006 (^*Pk*^PhaG Q45R G142V vs. ^*Pk*^PhaG), **P = 0.004 (^*Pk*^PhaG Y138F vs. ^*Pk*^PhaG), *P = 0.02 (^*Pk*^PhaG Q45R vs. ^*Pk*^PhaG) and *P = 0.03 (^*Pk*^PhaG G142V vs. ^*Pk*^PhaG) were analyzed based on two-tailed student *t* test assuming unequal variances. Source data underlying B are provided as a Source Data file.

We initiated a protein engineering study to seek variants with enhanced activity altered and if possible altered product specificity. We constructed an error-prone PCR library of ^*Pk*^*phaG* ORFs and screened for the ability to complement a lipoic acid auxotrophy; this approach was previously used to isolate C_8_-specific thioesterases with enhanced *V*_max_^14^. *E. coli* strains lacking LipB require supplementation of lipoic acid or octanoic acid in the media to enable the formation of active pyruvate dehydrogenase and aerobic growth on glucose. We constructed a lipoic acid auxotrophic strain, *E. coli* CM23-Δ*lipB*, that also lacks β-oxidation genes and many fermentation pathways. To link the octanoic acid selection to PhaG activity, we added heterologous enzymes to convert the 3-hydroxyoctanoyl-CoA to octanoic acid (Fig. 4A). These enzymes include ^*Pk*^PhaG, ^*Pa*^PhaJ3, a *Treponema denticola* trans-enoyl-CoA reductase (^*Td*^TER)^8^ and a *Mycobacterium* sp. acyl-CoA thioesterase ^*Ma*^TesB A197D (referred as to ^*Ma*^TesB*)^45^ (Fig. 4A). Purified *Mycobacterium avium* ^*Ma*^TesB* has been shown to hydrolyze octanoyl-CoA and generate octanoic acid in vitro^45^. We neglected the octanoic acid synthesis activities by endogenous *E. coli* acyl-CoA thioesterase because ^*Ec*^TesB generally has activities toward longer chain acyl-CoA (>C10)^41^ and ^*Ec*^YciA has activities toward shorter chain acyl-CoA (<C_8_)^46,47^. The base strain, expressing the wild-type ^*Pk*^*phaG*, produced ~20 mg/L octanoic acids after 48 h, whereas the corresponding strain without ^*Pk*^PhaG produced <1 mg/L octanoic acid. In order to reduce the baseline octanoic acid titer, we subcloned ^*Pk*^*phaG* onto a low-copy vector (pBTRCK)^8^ and ^*Ma*^tesB* onto a high copy number vector pTRC99a. The latter was performed to ensure that octanoic acid production would be limited solely by PhaG activity. After tuning the copy number of ^*Pk*^*phaG and* ^*Ma*^tesB*, E. coli CM23-Δ*lipB* harboring pTRC99a-^*Ma*^tesB*-^*Td*^*TER* + pACYC-^*Pa*^*phaJ3* + pBTRCK-’^*Pk*^*phaG*’ plasmids produced ~7 mg/L octanoic acids after 48 h. This strain was used to perform selections of the error-prone PhaG library.

In the first-round of mutagenesis, hundreds of colonies appeared three days after plating on MOPS-glucose minimal agar containing 20 μM isopropyl β-d-1-thiogalactopyranoside (IPTG). On day 4, we picked ~180 of the largest colonies and quantified the octanoic acid titer from individual liquid cultures grown in Clomburg liquid media containing 20 g/L glycerol. We found 17 ^*Pk*^PhaG variants increased octanoic acid titer (3.3–16.3-fold) and total fatty acid titer (1.8–8.3-fold) relative to the parent ^*Pk*^PhaG (Supplementary Figures 3–5 and Supplementary Method 2). The 17 improved ^*Pk*^PhaG variants contained a total of 28 point mutations that we recreated individually. We co-expressed each ^*Pk*^PhaG variant from the high copy number pTRC99a-’^*Pk*^*phaG*’-^*Td*^*TER* vector with pACYC*-*^*Pa*^*phaJ3* + pBTRCK-^*Ma*^tesB* in *E. coli* CM23. Each strain was cultured for 48 hrs at 30 °C and samples were harvested for fatty-acid quantification. We found that variants containing 6 of the 28 single point mutations (e.g., Q45R, R66H, H76Y, Y138F, G142V, Q277X) increased octanoic acid titer more than twofold over strains expressing the parent ^*Pk*^PhaG (Supplementary Figure 6). Among these, three individual ^*Pk*^PhaG mutants Q45R, G142V and Y138F strains had 1.9-, 1.8-, and 1.5-fold higher total fatty acid titers than strains expressing wild-type ^*Pk*^PhaG.

We constructed all four possible combinations of Q45R, G142V, and Y138F mutations in a vector pTRC99a-’^*Pk*^*phaG*’-^*Td*^*TER* and repeated analogous fatty-acid production experiments. The best variant, ^*Pk*^PhaG Q45R G142V, produced 1.1 g/L of C_8_–C_14_ free fatty acids, a 4.0-fold increase compared with the original ^*Pk*^PhaG (Fig. 4B). The fatty-acid pool contained 41% tetradecanoic acid, 22% dodecanoic acid, 11% decanoic acid, and 26% octanoic acid, similar to the original ^*Pk*^PhaG-expressing strain. This indicates that the increased production of octanoic acid was due to a general increase in activity, not selectivity.

**Fig. 5:**
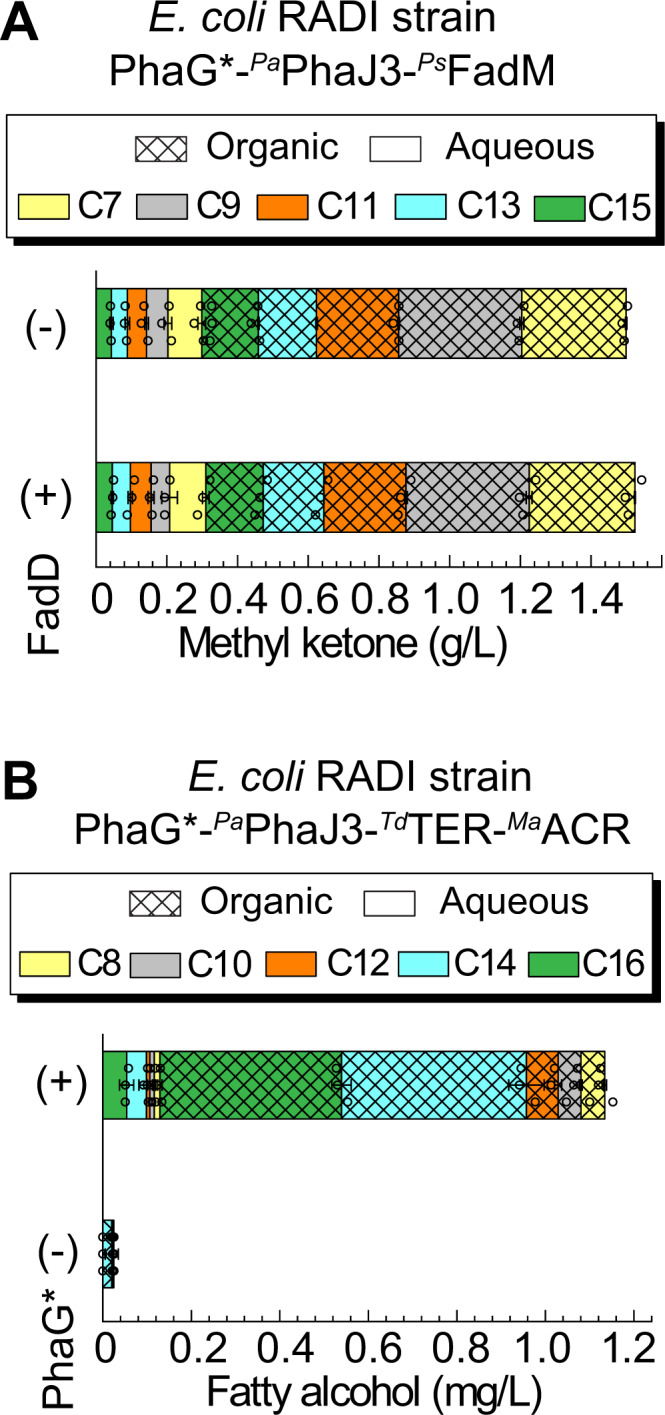
Methyl ketone and fatty alcohol production using PhaG*. **A** Methyl ketone production by *E. coli* RAI or RADI harboring pTRC99a-^*Pk*^phaG*-^*Ps*^*fadM* + pACYC-^*Pa*^*phaJ3* plasmids. **B** Fatty alcohol production by *E. coli* RADI harboring pTRC99a-^*Pk*^phaG*-^*Td*^*TER* + pACYC-^*Pa*^*phaJ3* + pBTRCK-^*Ma*^*ACR*. The corresponding negative control harbored a pTRC99a empty vector in place of the PhaG* vector. All cells were grown in 50 mL of Clomburg media containing 20 g/L glycerol, 20% (v/v) dodecane, and 1 mM IPTG at 30 °C for 48 hrs (*n* = 3 biologically independent samples). The shadings and non-shadings in graphs represented products in the dodecane organic layer and aqueous phase, respectively. All data represent the mean ± s.d. of biological triplicates. Source data underlying A, B are provided as a Source Data file.

We next repeated the experiments described in Fig. 3 to confirm that the more-active ^*Pk*^PhaG* (referred as to ^*Pk*^PhaG Q45R G142V) variant was not exceeding the downstream FadB and PhaJ activities. Strains expressing ^*Pa*^PhaJ3, which was present in all protein engineering experiments, and ^*Pa*^PhaJ1 produced the highest fatty acid titers (Supplementary Figure 7) and more than any other alternative dehydratase. Similar to the observation in Fig. 2C, deletion of FadD did not substantially affect methyl ketone titer (Fig. 5A) generated by the enhanced ^*Pk*^PhaG*, suggesting that the modified enzyme does not have enhanced thioesterase activity. Note, these experiments were performed with a FadM thioesterase from *P. sneebia* that we found had higher activity than *E. coli* FadM^4^.

### Benchmarking PhaG-driven oleochemical production

Next, we benchmarked PhaG-driven production of methyl ketones and fatty alcohols as model oleochemical products. To produce methyl ketones, we cultured *E. coli* RADI harboring pTRC99a-^*Pk*^phaG*-^*Ps*^*fadM* and pACYC-^*Pa*^*phaJ3*. To produce fatty alcohols, we cultured strain *E. coli* RADI harboring pTRC99a-^*Pk*^phaG*-^*Td*^*TER*, pACYC-^*Pa*^*phaJ3*, and pBTRCK-^*Ma*^*ACR*. A bi-functional alcohol forming acyl-CoA reductase ^*Ma*^ACR from *M. aquaeolei* VT8 was chosen because of its successful use in prior studies^8^. Each strain was cultured for 72 hr at 30 °C in shake flasks containing rich glycerol media. The cultures produced 1.5 g/L total methyl ketone and ~1.1 g/L total fatty alcohol from 20 g/L glycerol (Fig. 5). The chain length distribution of each product was different. Methyl ketones were evenly distributed across 8–16-carbon chain lengths, whereas, fatty alcohols were dominated by 14- and 16-carbon alcohols. The titer and yield of fatty acids, methyl ketones, and fatty alcohols from the PhaG-dependent strategies are comparable to values reported in the literature using thioesterase strategies or reverse β-oxidation strategies to produce products in shake flasks (Table 1).

**Table 1:** Reported titers and yield of oleochemical production using established thioesterase-dependent strategies or developed PhaG-dependent strategies in this work.

Strategies	Products	Titer (g/L)	Yield	% TY^*^	Fermentation
Thioesterase					
^Cp^FatB^4^	2-heptanone,	4.4 g/L,	0.028 mol/mol consumed glycerol,	9%	Fed-batch, bioreactor
	2-nonanone,	3 g/L,	0.018 mol/mol consumed glycerol,	8%	
	2-undecanone	0.34 g/L	0.0008 mol/mol consumed glycerol	0.4%	
^Cp^FatB^18^	1-octanol	1.3	0.046 mol/mol glycerol	17.4%	Batch, shake flask,
^Cp^FatB^14^	octanoic acid	1.7	0.054 mol/mol glycerol	17.4%	Batch, shake flask,
BTE^52^	C_12_-C_14_ FAOH	1.6	0.109 mol/mol glucose^b^	34.8%	Fed-batch, bioreactor
^Ec^TesA^15^	C_8_-C_16_ FFA	0.5	0.044 mol/mol glycerol	14%	Batch, shake flask
^Ec^TesA*^15^	C_8_-C_16_ FFA	0.67	0.108 mol/mol glycerol	34%	Batch, shake flask
^Ec^TesA^53^	C_11_-C_17_ MK	9.8	0.154 mol/mol glucose^c^	53%	Fed-batch, bioreactor
^Ec^TesA^54^	C_11_-C_17_ MK	5.4	0.033 mol/mol glucose^c^	11%	Fed-batch, bioreactor
r-BOX					
r-BOX^6^	C_6_-C_16_ FAOH	1.8	0.2 mol/mol consumed glucose	65%	Fed-batch, bioreactor
r-BOX^10^	C_4_-C_10_ FFA	4.7	0.25 mol/mol consumed glucose^d^	68%	Fed-batch, bioreactor
PhaG					
^*Pk*^PhaG	C_8_-C_14_ FFA	1.2	0.063 mol/mol glycerol^a^	21%	Batch, shake flask
^*Pk*^PhaG	C_8_-C_16_ FAOH	1.1	0.024 mol/mol glycerol^b^	15%	Batch, shake flask
^*Pk*^PhaG	C_7_-C_15_ MK	7.2	0.049 mol/mol glycerol^c^	31%	Fed-batch, bioreactor

^a^Dodecanoic acid M.W. was used as an average M.W. of the fatty acid mixture.

^b^1-tetradecanol M.W. was used as an average M.W. of the fatty alcohol mixture.

^c^2-tridecanone M.W. was used as an average M.W. of methyl ketone mixture.

^d^Octanoic acid M.W. was used as an average M.W. of the fatty acid mixture.

*Percentage of the theoretical yield.

**Fig. 6:**
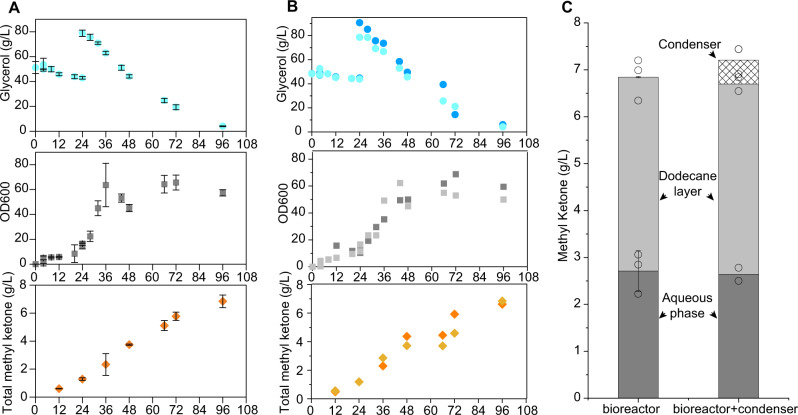
Fed-batch fermentation for methyl ketone production. Time course of glycerol consumption, OD_600_, and methyl ketone titer from fed-batch bioreactor cultures (*n* = 3 biologically independent samples). **A** and bioreactor coupling a condenser (*n* = 2 biologically independent samples) **B** using *E. coli* RADI harboring pTRC99a-^*Pk*^phaG*-^*Ps*^*fadM* and pACYC-^*Pa*^*phaJ3* plasmids. **C** Evaluation of methyl ketone concentration from samples in the aqueous phase (dark gray), dodecane layer (light gray), and condenser (shading) after 96 h fermentation. All data represent the mean ± s.d. of biological triplicates. Source data underlying **A**–**C** are provided as a Source Data file.

To further improve methyl ketone titers, we performed discontinuous fed-batch cultivations in a stirred bioreactor by adding media pulses after cells reached high cell densities. Enhanced aeration in bioreactors can lead to loss of volatile oleochemical products in the off-gas. Therefore, we added a 20% dodecane overlay to the culture^4^ to trap products in the reactor and designed a gas trap to recover products from the off-gas. Details of off-gas methyl ketone capture and ASPEN analysis can be found in the Supplementary information file (Supplementary Figure 8 and Supplementary Method 3). After 96 hrs of cultivation in reactors lacking an off-gas scrubber, cells consumed 90.0 g/L of glycerol, reached an OD_600_ ~60, and produced 6.8 g/L total methyl ketone (Fig. 6A). In a separate experiment, we bubbled the bioreactor off-gas through a jacketed gas dryer filled with dodecane maintained at 5 °C. After 96 hrs of induced cultivation, cells reached a density of OD_600_ ~60, consumed 95.2 g/L glycerol, and produced 6.7 g/L total methyl ketone (Fig. 6B). At the endpoint of the cultivation, we observed 3.4 g/L methyl ketone contained in the condensed dodecane phase of the off-gas trap, corresponding to an additional 0.51 g/L (per L of culture volume) of total methyl ketone captured from the bioreactor (Fig. 6C).

This study demonstrated that PhaG is capable of supporting high-flux to three medium-chain oleochemical products, fatty acids, fatty alcohols, and methyl ketones. Through bioprospecting, we identified a PhaG from *P. koreensis* that demonstrated higher activity when producing methyl ketones. Random mutagenesis of ^*Pk*^PhaG produced 17 enhanced PhaG variants that were isolated by complementing lipoic acid auxotrophy. The improved activity was demonstrated by 3–16-fold more octanoic acid being produced when the PhaG variants were co-expressed in a cell designed to direct flux to octanoic acid (co-expressing TesB, PhaJ3, and Ter). Reconstitution of the individual point mutations led to the creation of a double mutant, ^*Pk*^PhaG Q45R G142V, that showed 4.0-fold higher activity relative to the parent enzyme. Finally, we demonstrated the production of 1.1 g/L C_8_–C_14_ free fatty acids, 1.5 g/L C_7_–C_15_ methyl ketones, and 1.1 g/L C_8_–C_16_ fatty alcohols in shake flasks and 7.2 g/L of methyl ketones in a fed-batch. These results demonstrate that PhaG is a viable alternative strategy that should be considered for oleochemical production. The PhaG-dependent strategy has the potential to achieve higher theoretical yields compared with the well-established thioesterase route. However, this impact, saving one ATP per product, is not likely to be observed until cells approach theoretical limits. Given the current state of the field, additional work is needed on both fronts. Our work motivates continued strain development as well as additional protein engineering^15,48,49^ to narrow the PhaG product profile and further increase activity.

## Methods

### Bacterial strains, plasmids, oligonucleotides, and reagents

All bacterial strains used in this study are listed in Supplementary Data 1. Q5 DNA polymerase and Monarch^®^ PCR and DNA Cleanup Kit were purchased from New England Biolabs (Ipswich, MA). Oligonucleotide primers and gBlocks were synthesized by Integrated DNA Technologies (IDT), Inc. (San Diego, CA). Chemicals including fatty acid, fatty alcohol, and methyl ketones were purchased from Sigma-Aldrich (St. Louis, MO).

*E. coli* DH5α strains were used for plasmid amplification and DNA assembly. *E. coli* RABIJ (MG1655 *ΔaraBAD ΔfadR ΔfadA ΔfadB ΔfadI ΔfadJ*) and *E. coli* CM23 (MG1655*ΔaraBAD ΔFadABIJDRE ΔldhA ΔackApta ΔadhE ΔpoxB ΔfrdABCD ΔydiO ΔatoC*) were created as part of prior studies^11,26^. *E.coli* RAI (MG1655 *ΔaraBAD ΔfadR ΔfadA ΔfadI*) and RADI (MG1655 *ΔaraBAD ΔfadR ΔfadA ΔfadI ΔfadD*) strains were used in those experiments presented in Figs. 2B–D, 3B, 5A, B, 6A, B. *E. coli* CM23 strains were used in those experiments presented in Fig. 4B.

### Plasmid and strain construction

All plasmids used in this study are summarized in Supplementary Data 1. Plasmid maps are available in Source Data file folder. All plasmids were constructed by Gibson assembly (New England Biolabs). The chromosomal deletion of *fadD* and *lipB* were performed using a combination of lambda red recombination and CRISPR/Cas9-mediated selection^4,8,14,18^. All cloned sequences and gene deletions were confirmed by Sanger sequencing performed by Functional Biosciences (Madison, WI). Constructs expressing FadB^4,8^ and PhaJ homologs were obtained from prior studies^44^.

### Oleochemical production and quantification

All oleochemical production studies were performed by growing *E. coli* strains at 30 °C in Clomburg medium containing 20 g/L glycerol, the appropriate antibiotics (Carbenicillin—100 μg/mL; kanamycin—50 μg/mL; chloramphenicol—34 μg/mL), and IPTG for induction as indicated. Pre-cultures for each experiment were prepared by inoculating 5 mL LB media (+antibiotics) with a single colony and incubating overnight at 30 °C with shaking at 250 r.p.m. A 2.5 vol% inoculum was transferred into production flasks with a starting OD_600_ 0.1. Fatty alcohol and methyl ketone production cultures were supplemented with 10% (v/v) dodecane to provide a product sink. Samples from each culture were extracted after 72 h incubation at 30 °C.

Fed-batch fermentation was performed using a 1-L Infors Multifors bioreactor. Overnight pre-cultures were inoculated to an initial OD_600_ of 0.05 into a bioreactor containing 500 mL Clomburg medium with ~50 g/L glycerol. The bioreactor was operated at the following conditions: the temperature was controlled at 30 °C post induction, airflow was 1.5 L/min, stirrer rate was varied between 250 r.p.m. and 1000 r.p.m. to control dissolved oxygen at a value of 30%, pH was maintained at 7.0 using 2 M sulfuric acid and 2 M ammonia hydroxide. When the OD_600_ reached ~1.0, IPTG was added to achieve a final concentration of 1 mM and 100 mL dodecane was fed in the bioreactor. At 24 h of post induction, ~100 mL 5*×* concentrated Clomburg media containing 500 g/L glycerol was one-time bolus-fed into the bioreactor and fermentation terminated 96 h post induction. Measurements of methyl ketone, glycerol, optical density, and CO_2_ evolution were recorded for 96 h total.

The bioreactor outlet gas stream was directed through a chilled organic absorber to capture methyl ketone vapors stripped from the culture broth. The absorber was composed of a jacketed glass gas dryer with a ceramic sparge distributing the off-gas into 100 mL of chilled dodecane. The temperature of the absorber was maintained at 5 °C by an external water cooler. Methyl ketone data were taken at 96 h from the dodecane, and endpoint methyl ketone capture was taken from both the collected water and remaining dodecane 96–101 h after inducing the culture. A schematic of the absorber is described in more detail in Supplementary Figure 7.

To determine the methyl ketone and fatty alcohol concentration in the distinct organic or aqueous phases, 50 mL of cell culture was centrifuged at 4500 *×* *g* for 10 min and 0.5 mL samples from the dodecane layer and 2.5 mL samples from the aqueous phase were collected and evaluated separately. Fatty acids were extracted from culture according to an acid-based esterification method^14,15^. Fatty alcohols and methyl ketones were extracted from culture into *n*-hexane^4,8^. Fatty acid and methyl ketone species were separated using an Agilent RTX-5 column and fatty alcohol species were separated using Agilent DB-Fatwax column (Santa Clara, CA). Oleochemicals were quantified by comparing GC-FID peak areas against standard curves prepared with commercial standards.

### Mutagenesis of PhaG

A mutagenic PhaG library was constructed by error-prone PCR using GeneMorph II from Agilent (Senta Clara, CA) with a low mutation frequency (0–4.5 mutations/kb). The plasmid backbone (pBTRCK) was PCR-amplified using a high-fidelity DNA polymerase Q5 from New England Biolabs (Ipswich, MA). The library was assembled using an isothermal assembly method^50^. Primers used in the creation of the library contained the start and stop codons in order to prevent mutation of them.

The sequence of improved PhaG variants was obtained by Sanger sequencing of colony PCR products made by high-fidelity Q5 DNA polymerase. To re-introduce single point mutations, we amplified ^*Pk*^PhaG with mutagenic primers and subcloned the fragments into pTRC99A-’^*Pk*^*phaG*’-^Td^*TER* by an isothermal assembly method^51^. The resulting plasmids were transformed into *E. coli* CM23 harboring pBTRCK-^*Ma*^tesB* and pACYC-^*Pa*^*phaJ3* plasmids.

### Lipoic acid selection

3-Hydroxyoctanoyl-CoA producing variants of PhaG were isolated using a lipoic/octanoic acid selection strategy^14^. In brief, Gibson assembly reaction mixtures (2 μL) containing a PhaG library (pBTRCK-^*Pk*^phaG*) was transformed into 100 μL of electrocompetent *E. coli* CM23-*ΔlipB* (CM23 strain, *ΔlipB*) strain harboring pTRC99a-^*Td*^*TER*-^*Ma*^tesB* + pACYC-^*Pa*^*phaJ3*. Transformants were plated on MOPS minimal media agarose plates containing 0.2% glucose, 20 μM IPTG, and kanamycin, chloramphenicol, and carbenicillin to maintain and induce plasmids^14^. Cells were rescued growth and appeared on the selection plates after 3 days of incubation. Transformants were patched onto LB plates for archiving and secondary screening in octanoic acid production studies.

### Statistics

We used instrument software to collect and analyze most experiments. Shimadzu Labsolutions (Long Beach, CA) was used for GC and HPLC data analysis. The fluorescence intensity of YFP and RFP was detected by the Tecan 200 Plate reader software 3.9.1.0. (Männedorf, Switzerland). Error bars indicate standard deviations from three biological replicates. All *P* values were generated from a two-tailed Student’s *t* test using Microsoft Excel 2016 (Microsoft Corporation, USA).

### Reporting summary

Further information on research design is available in the Nature Research Reporting Summary linked to this article.

## Supplementary information

Supplementary Information

Description of Additional Supplementary Files

Supplementary Data 1

Supplementary Data 2

Supplementary Data 3

Supplementary Data 4

Reporting Summary

## Data Availability

The authors declare that all data supporting the findings of this study are available within the paper (and its Supplementary Information files). All strains and plasmids information are available in Supplementary Data 1. The additional reactions and metabolites used in Oleo_iML1515 than iML1515 are available in Supplementary Data 2 and 3. All plasmid maps including annotations of oligonucleotide and gene sequences are available in Supplementary Data 4. Source data are provided with this paper.

## Source data

Source Data

## Code availability

All codes for calculating theoretical yields are available at the GitHub repository [https://github.com/Pfleger-Lab/Metabolic-Modeling---Yield-Analysis-of-PhaG].
