# Supplementary material for: Metabolic engineering strategies to produce medium-chain oleochemicals via acyl-ACP:CoA transacylase activity
Source: Nat Commun. 2022 Mar 25;13:1619. doi: 10.1038/s41467-022-29218-3 (PMC8956717; doi:10.1038/s41467-022-29218-3)
Supplement: Supplementary file 1 — Supplementary Information [file 41467_2022_29218_MOESM1_ESM.pdf]

Metabolic engineering strategies to produce medium-chain oleochemicals *via* acyl-ACP:CoA transacylase activity

Yan *et al*.

Supplementary Method 1. Genome scale metabolic model calculations

Maximum theoretical yields were calculated via flux balance analysis of a modified version of the iML1515 genome-scale metabolic model of *Escherichia coli* utilizing the cobrapy python package[1]. Mass and charge balanced reactions catalyzed by PhaG were added to the iML1515 model. Lower bounds for biomass production and non-growth associated maintenance requirements were set to 0 mmol gDW^{-1} h^{-1}, and each theoretical yield was calculated as the ratio of maximized product flux to glycerol uptake flux (Supplementary Table 1). Flux was constrained to each described pathway through simulated gene knockouts, or individual reaction constraints. An IPython notebook containing the script used is included in "PhaG yield analysis.ipynb" of GitHub repository [https://github.com/Pfleger-Lab/Metabolic-Modeling---Yield-Analysis-of-PhaG], and the modified iML1515 model is included in "Oleo_iML1515.xml" of the same GitHub repository.

Supplementary Method 2. Evaluate PhaG expression levels

To investigate why titers were increased in these strains, we examined PhaG abundance using a translational coupling circuit linking RFP to PhaG[2]. We compared expression of five PhaG variants (WT, LM1, LM7, LM9 and LM14) by proxy using RFP fluorescence generated by the translationally coupling cassette. When YFP was cloned in place of PhaG, RFP fluorescence was proportional to YFP fluorescence over a range of IPTG (inducer) concentrations (Supplementary Figure 5). PhaG variants LM9, LM14, and WT demonstrated equivalent fluorescence values across a range of IPTG concentrations. PhaG variant LM7 generated slightly more RFP fluorescence, indicating it may be present at higher concentrations. Conversely, PhaG variant LM1 produced

~60% of the RFP fluorescence of the WT. Decreased expression may be due to the occurrence of a rare codon CGG at point mutation Q45R. CGG codons have been shown in other studies to be a rate-limiting factor for translational efficiency due to its low abundance tRNA supply in *E. coli*[3,4]. We estimated specific PhaG activity by normalizing the sum of observed C_8-C_{14} FAME titer to RFP fluorescence (proxy for protein abundance). These estimates indicate the LM1 and LM14 variants are 7.4- and 8.8-fold more active than wild-type PhaG (Supplementary Figure 5).

Supplementary Method 3. Off gas methyl ketone capture and ASPEN analysis

In effort to capture methyl ketones lost from shake flask experiments, we constructed a chilled condenser[4] to capture product lost in the off-gas stream of our bioreactors. This condenser provided only a modest increase in methyl ketone titer, suggesting that either there is not a significant loss of methyl ketones over time, or that the condenser was unable to condense the lost product. Aspen Plus V11 was used to estimate the feasibility of 2-heptanone capture using a condenser. The UNIFAC liquid-liquid (UNIF-LL) property method was used to model the liquid-liquid equilibrium between water, dodecane and 2-heptanone. The simulation consisted of a three-outlet flash drum and a two-outlet flash drum. The three-outlet flash modeled the bioreactor and served to separate the vapor, aqueous (water), and organic (dodecane) product/outlet streams. The operating pressure for all units was 1 bar and the operating temperature for the two-outlet flash was 5°C. The flowrates of water, dodecane, and air in the feed were 500 g/min, 133 g/min, and 1320 g/min, respectively to represent the ratio of these species in the bioreactor experiment. The flowrate of 2-heptanone was arbitrarily set to 1.0 g/min, as the focus of this analysis was the fractional recovery of 2-heptanone, which was not found to change significantly at different

flowrates. The mass fraction of 2-heptanone recovered in the liquid outlet of the flash drum was 3.8%. The simulation was repeated to determine if the recovery could be improved by using an organic gas absorber instead of a condenser. The vapor product stream of the three-outlet flash drum was feed into a cooler (heat exchanger), cooled to 5°C, then fed into an absorber (modeled by a two-stage column). A secondary dodecane stream at a flowrate of 133 g/min was (also) fed into the absorber. The fractional recovery of 2-heptanone in the dodecane product stream was at least 90%. The recovery was not found to change significantly at different 2-heptanone initial flowrates. These results suggest that the recovery of 2-heptanone from the bioreactor off-gas could be significantly improved if an absorber was used instead of a condenser.

To implement a gas absorber, the bioreactor off gas was fed directly into a jacketed gas dryer filled with 100mL of dodecane and chilled to 5°C with an external Fisher Scientific Isotemp water cooler (Supplementary Figure 8). Over time water evaporated from the reactor collected in the absorber varying from 50-100 mL. Water collected was removed as needed to prevent dodecane overflow and combined and analyzed for methyl ketone concentration at the completion of the bioreactor run. Total methyl ketones in the absorber were calculated from a sample of dodecane and the collected water at the end of the fermentation and added to the total bioreactor titer.

Supplementary Table 1. Theoretical yields of oleochemicals synthesis from glycerol as a carbon source using the thioesterase route or transacylase route.

	TE	PhaG
methyl ketones		
2-heptanone	0.3	0.318
2-nonanone	0.236	0.247
2-undecanone	0.194	0.202
2-tridecanone	0.165	0.17
fatty alcohols		
1-octanol	0.267	0.276
1-decanol	0.215	0.221
1-dodecanol	0.18	0.184
1-tetradecanol	0.154	0.157
fatty acids		
octanoic acid	0.294	0.294
decanoic acid	0.232	0.232
dodecanoic acid	0.188	0.188
tetradecanoic acid	0.163	0.163

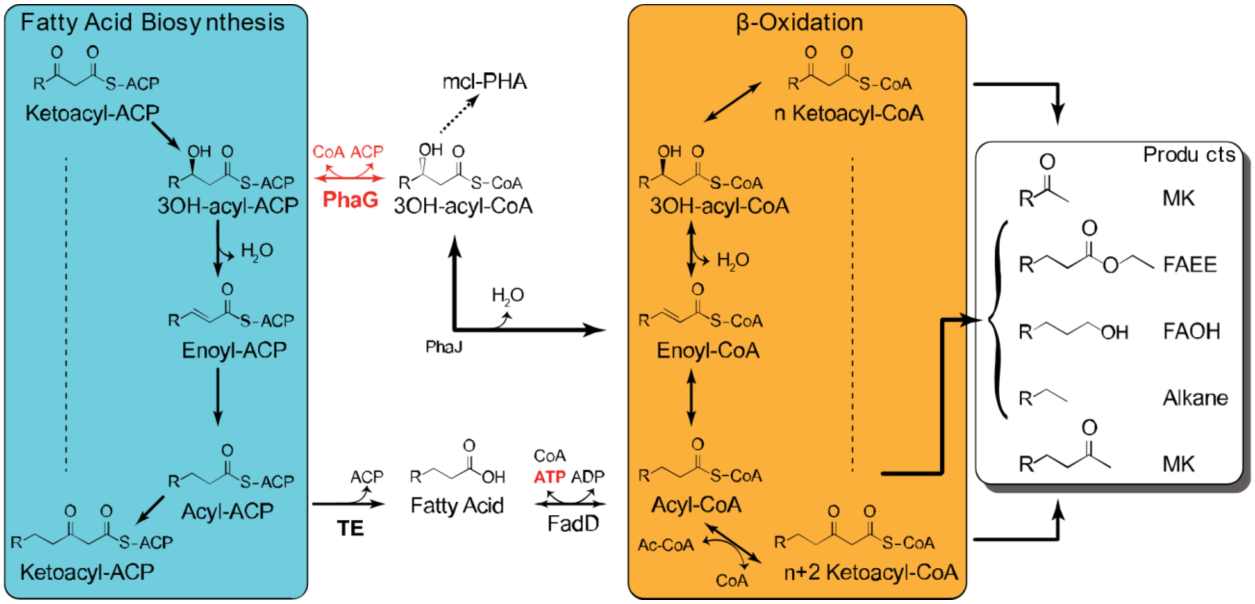

Supplementary Figure 1. Detailed thioesterase and PhaG biosynthetic pathways for medium-chain oleochemical production.

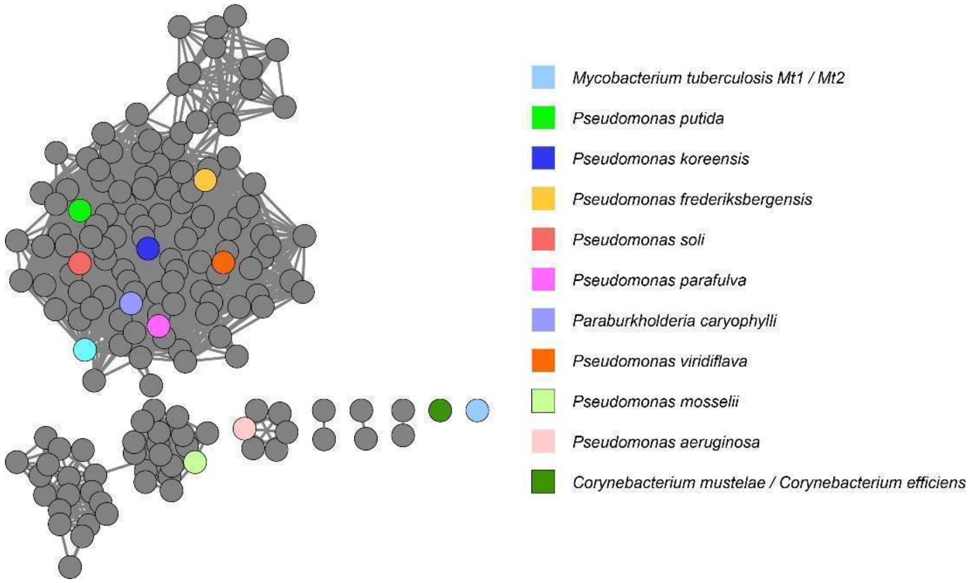

		PpuPhaG	PmPhaG	PsPhaG	PpaPhaG	PcPhaG	PkPhaG	PvPhaG	PfPhaG	PaPhaG	CmPhaG	CePhaG	Mt1PhaG	Mt2PhaG
Pseudomonas putida	PpuPhaG	100	88	87	83	78	68	66	62	60	28	25	22	24
Pseudomonas mosselii	PmPhaG	88	100	95	86	79	70	66	64	60	35	26	24	26
Pseudomonas soli	PsPhaG	87	95	100	87	78	69	66	64	62	38	26	24	26
Pseudomonas parafulva	PpaPhaG	83	86	87	100	76	69	66	64	62	31	27	27	25
Paraburkholderia caryophylli	PcPhaG	78	79	78	76	100	72	73	64	62	22	23	22	32
Pseudomonas koreensis	PkPhaG	68	70	69	69	72	100	72	65	58	27	24	22	23
Pseudomonas viridiflava	PvPhaG	66	66	66	66	73	72	100	62	57	27	22	28	40
Pseudomonas frederiksbergensis	PfPhaG	62	64	64	64	64	65	62	100	62	34	25	33	31
Pseudomonas aeruginosa	PaPhaG	60	60	62	62	62	58	57	62	100	28	26	22	30
Corynebacterium mustelae	CmPhaG	28	35	38	31	22	27	27	34	28	100	26	22	27
Corynebacterium efficiens	CePhaG	25	26	26	27	23	24	22	25	26	26	100	32	25
Mycobacterium tuberculosis Mt1	Mt1PhaG	22	24	24	27	22	22	28	33	22	22	32	100	25
Mycobacterium tuberculosis Mt2	Mt2PhaG	24	26	26	25	32	23	40	31	30	27	25	25	100

Supplementary Figure 2. Similarity of PhaG homologs. (Top) Sequencing similarity map of PhaG homologs. (Bottom) The quantitative relationship between the sequences. Matrices show the pairwise percent amino acid identity multiple alignment of each enzyme homolog.

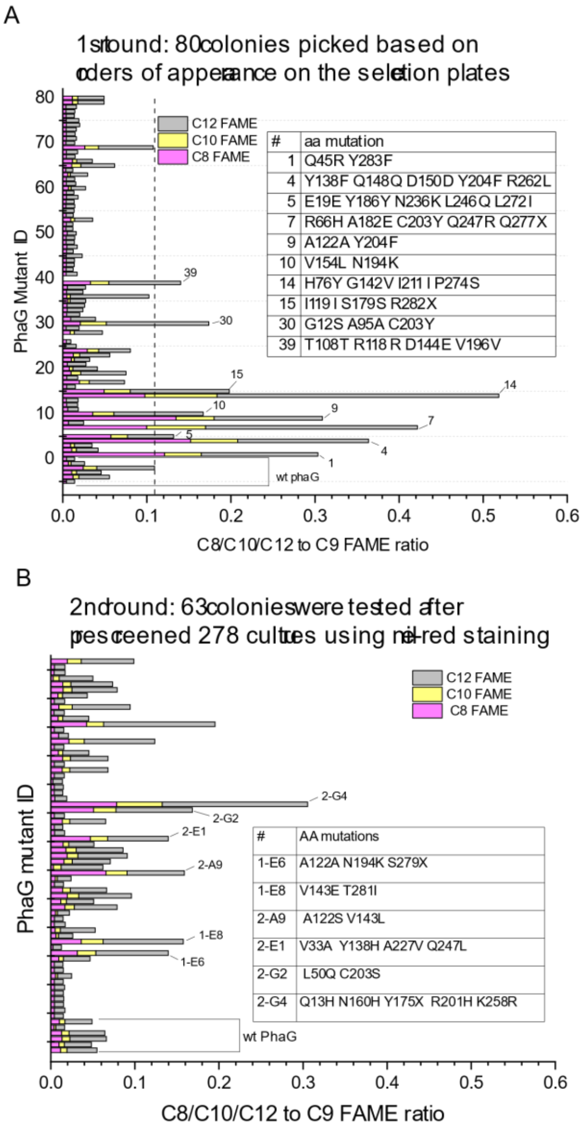

Supplementary Figure 3. Evaluation of fatty acid production of PhaG mutant library. (A-B) We took two strategies to pick colonies of hits. First, we picked 80 colonies that first appeared on selection plates on day 4. We cultured them for fatty acid production in Clomburg liquid media 20 g/L glycerol at 30°C for 48 hrs. In the second strategy, on day 5, we cultured another 287 colonies in 96-well plates in Clomburg liquid media 20 g/L glycerol at 30°C for 48 hrs. Culture supernatant was stained by 2 ul 1 mM nile-red. Fluorescence intensity of each culture was used to prescreen active PhaG enzymes compared to that of wild-type PhaG. Then, 64 cultures were further cultured in test tubes for fatty acid production. Source data are provided as a Source Data file.

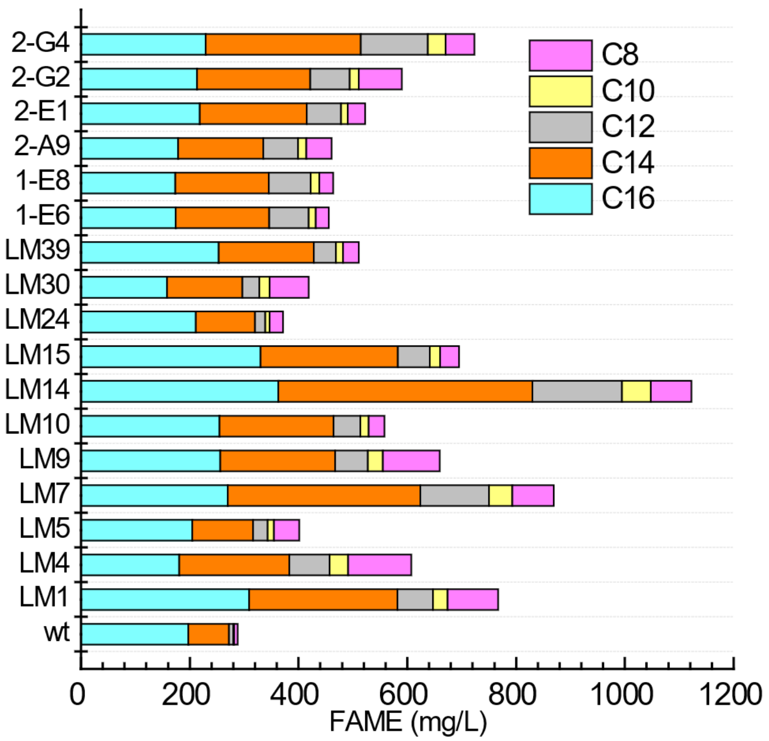

Supplementary Figure 4. Evaluation of fatty acid production of 17 beneficial PhaG mutants. See caption of Supplementary Figure 3 for further description. Source data are provided as a Source Data file.

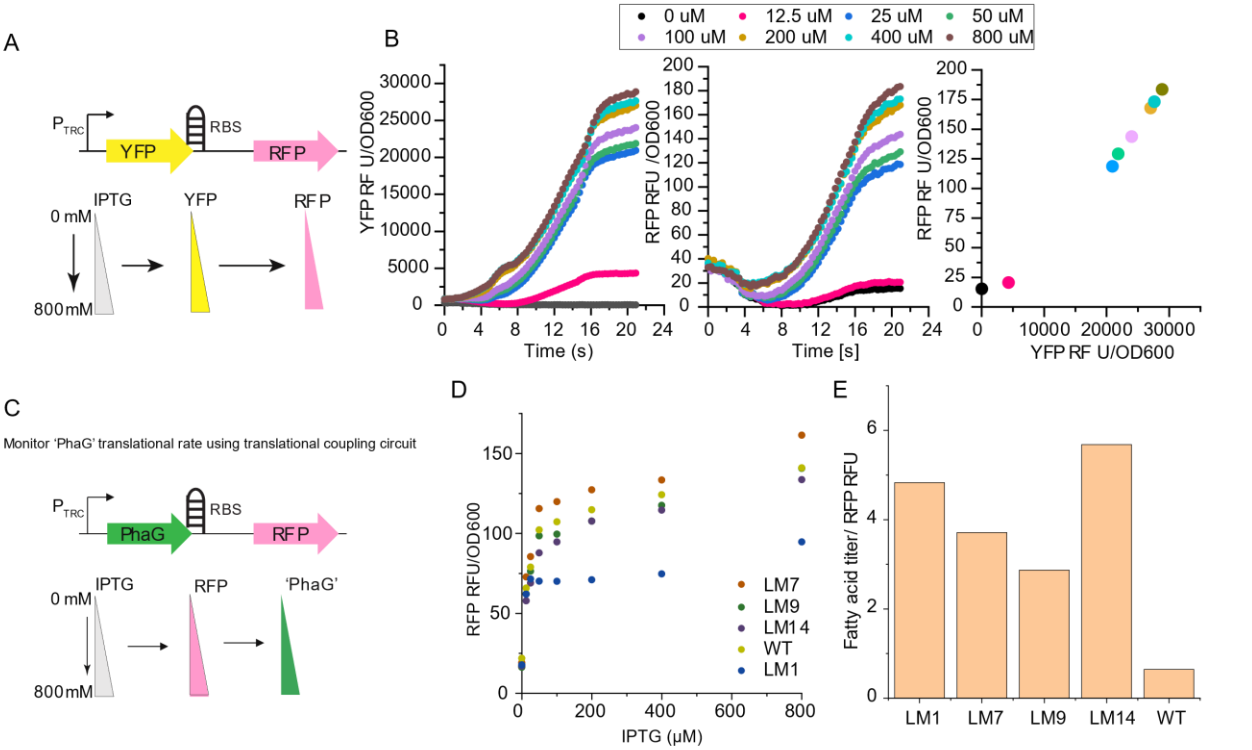

Supplementary Figure 5. Evaluation of PhaG Variant Expression Via Translational Coupling. (A) Scheme outlining translational coupling circuit linking RFP expression to YFP expression. YFP expression was titrated by induction of transcription with IPTG. (B) Time-course of YFP fluorescence intensity per OD600, RFP fluorescence intensity per OD600, and correlation of YFP and RFP fluorescence taken at the experiment end-point (48 hrs) were plotted. (C) Scheme outlining translational coupling circuit linking RFP expression to PhaG expression. PhaG variant expression was titrated by induction of transcription with IPTG. (D) End-point (48 hrs) RFP fluorescence intensity per OD600 (RFU/OD600) as a function of IPTG concentration was plotted for five *E. coli* DH5a strain harboring one of the p'PhaG'-RFP variants. (E) Evaluation of C8-C14 total fatty acid titer over PhaG abundance (RFP RFU/OD600). Source data are provided as a Source Data file.

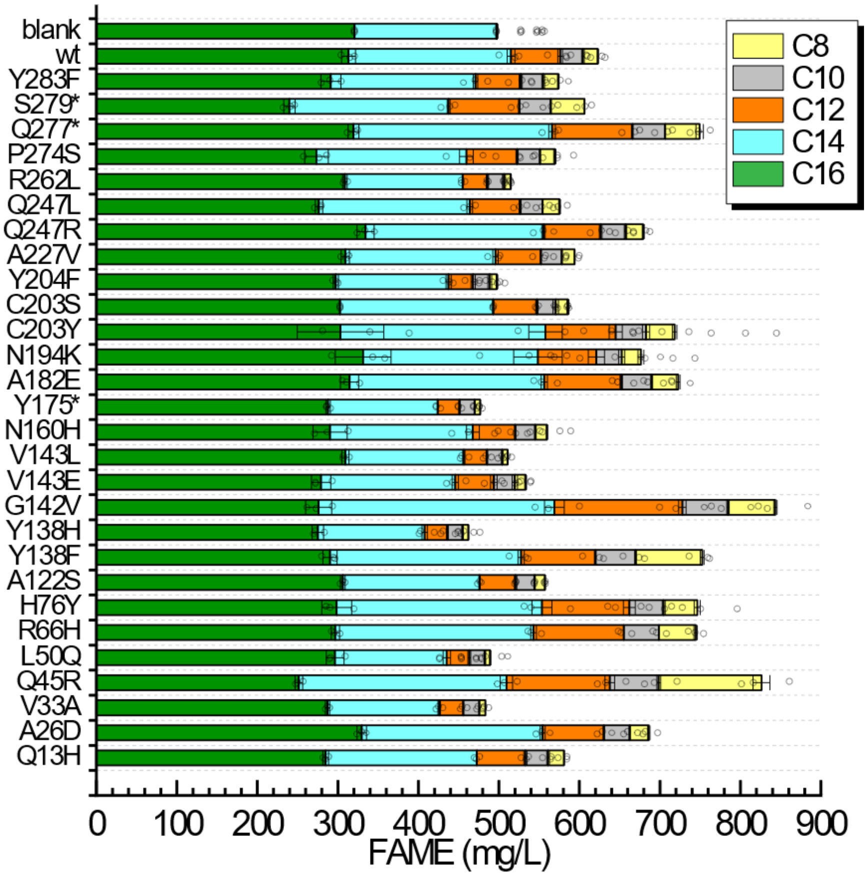

Supplementary Figure 6. Evaluate PhaG single point mutations activity. 28 ^{Pk}PhaG single point mutations were constructed and FAME profiles were evaluated using strain CM23 harboring pTRC99a-'^{Pk}*phaG*'-^{Td}*TER* + pACYC-^{Pa}*phaJ3* + pBTRCK-^{Ma}*tesB**. The strain was cultured in test tubes containing 5 mL Clomburg 20 g/L glycerol and 1 mM IPTG at 30°C for 72 hrs. All data represent the mean ± s.d. of biological triplicates. Source data are provided as a Source Data file.

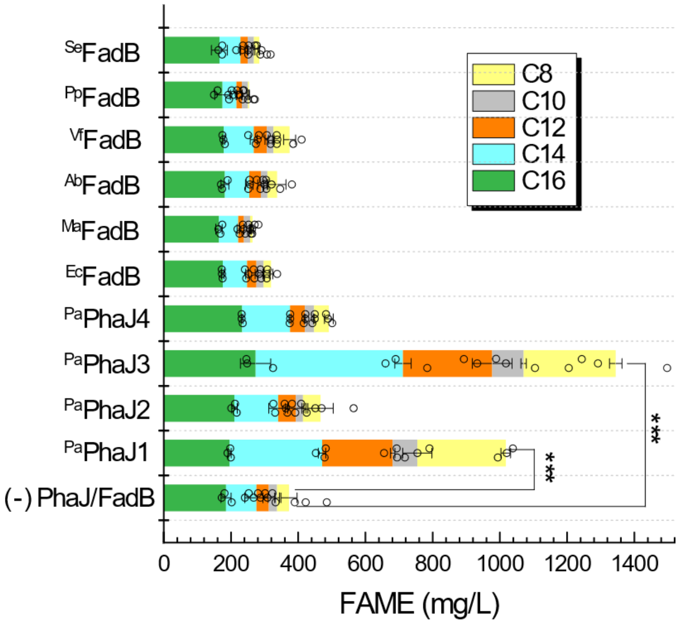

Supplementary Figure 7. Validating enzymes converting (R)-3-hydroxyacyl-CoA intermediates. In order to re-evaluate the effect of FadB and PhaJ activities on overall rate of the PhaG pathway with the more active ^{Pk}PhaG* (referred as to ^{Pk}PhaG Q45R G142V) variant, we cloned six FadB homologs and four PhaJ homologs in a pACYC vector, co-expressed each with pTRC99a-^{Td}*TER*-^{Pk}*phaG** and pBTRCK-^{Ma}*tesB** in strain CM23, and cultured them for fatty acid production (n=3 biologically independent samples). The strain expressing ^{Pa}PhaJ3 and ^{Pa}PhaJ1 produced the highest fatty acid titers, suggesting these enzymes have the highest activity for converting (*R*)-3-hydroxyacyl-CoA substrate to enoyl-acyl-CoA (Supplementary Figure7). We found that all FadB variants produced substantially lower titers compared to the analogous strains expressing ^{Pa}PhaJ3. This suggests that FadB has lower activity on recognizing (*R*)-3-hydroxyacyl-CoAs than ^{Pa}PhaJ3. This is not surprising since in *Pseudomonas*, FadB might evolve to lower its isomerization activity to avoid crosstalk between PHA biosynthesis and beta-oxidation. All data represent the mean ± s.d. of biological triplicates. ***P = 0.0001 (^{Pa}PhaJ3 vs. control) and ***P = 0.0001 (^{Pa}PhaJ1 vs. control) were analyzed based on student two-tailed t-test assuming unequal variances. Source data are provided as a Source Data file.

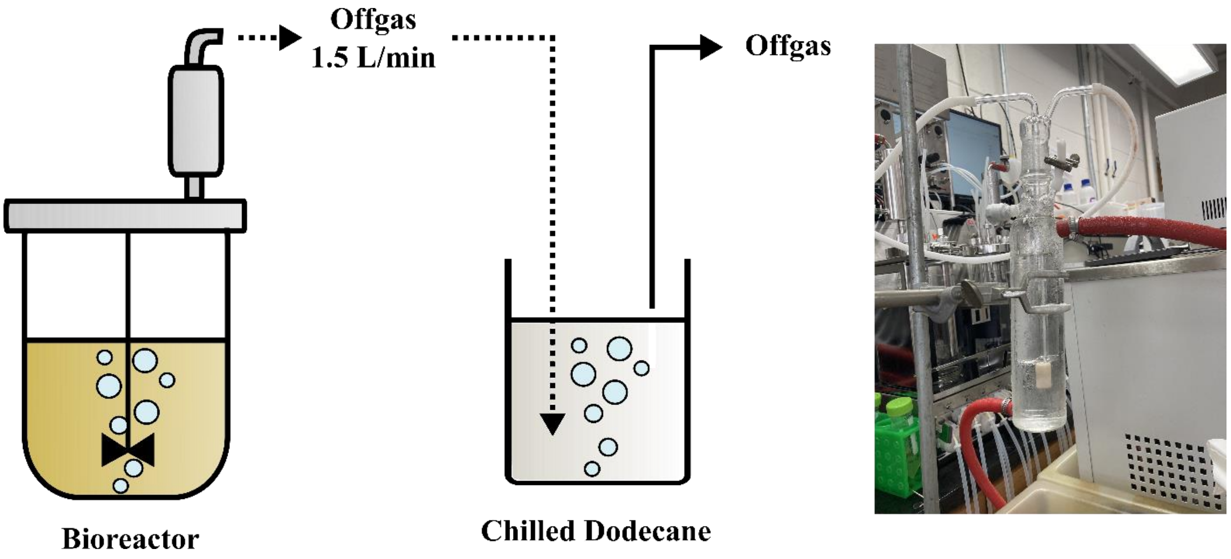

Supplementary Figure 8. Strategies of capture offgas methyl ketones. (A) (Left) Schematic of the bioreactor-absorber system, (B) (Right) Image of jacketed glass absorber filled with water and dodecane.

Supplementary references

1. Monk, J. M. *et al.* iML1515, a knowledgebase that computes *Escherichia coli* traits. *Nat. Biotechnol.* **35**, 904–908 (2017).
2. Mendez-Perez, D., Gunasekaran, S., Orler, V. J. & Pfleger, B. F. A translation-coupling DNA cassette for monitoring protein translation in *Escherichia coli*. *Metab. Eng.* **14**, 298–305 (2012).
3. Frumkin, I. *et al.* Codon usage of highly expressed genes affects proteome-wide translation efficiency. *Proc. Natl. Acad. Sci.* **115**, E4940–E4949 (2018).
4. Reis, M. d. Solving the riddle of codon usage preferences: a test for translational selection. *Nucleic Acids Res.* **32**, 5036–5044 (2004).
