# Supplementary material for: Metabolic engineering strategies to produce medium-chain oleochemicals via acyl-ACP:CoA transacylase activity
Source: Nat Commun. 2022 Mar 25;13:1619. doi: 10.1038/s41467-022-29218-3 (PMC8956717; doi:10.1038/s41467-022-29218-3)
Supplement: Supplementary file 2 — Description of Additional Supplementary Files [file 41467_2022_29218_MOESM2_ESM.pdf]

Description of additional Supplementary Files

File name: Supplementary Data 1

Description: Supplementary Table 2 Strains and plasmids used in this study

File name: Supplementary Data 2

Description: Additional metabolites added to iML1515 model

File name: Supplementary Data 3

Description: Additional reactions added to iML1515 model

File name: Supplementary Data 4

Description: Plasmid maps used in this study
