# Supplementary material for: Metabolic engineering strategies to produce medium-chain oleochemicals via acyl-ACP:CoA transacylase activity
Source: Nat Commun. 2022 Mar 25;13:1619. doi: 10.1038/s41467-022-29218-3 (PMC8956717; doi:10.1038/s41467-022-29218-3)
Supplement: Supplementary file 7 — Reporting Summary [file 41467_2022_29218_MOESM7_ESM.pdf]

nature portfolio

Corresponding author(s): Brian Pfleger

Last updated by author(s): Feb 24, 2022

Reporting Summary

Nature Portfolio wishes to improve the reproducibility of the work that we publish. This form provides structure for consistency and transparency in reporting. For further information on Nature Portfolio policies, see our Editorial Policies and the Editorial Policy Checklist.

Statistics

For all statistical analyses, confirm that the following items are present in the figure legend, table legend, main text, or Methods section.

n/a	Confirmed	
☐	☒	The exact sample size (*n*) for each experimental group/condition, given as a discrete number and unit of measurement
☐	☒	A statement on whether measurements were taken from distinct samples or whether the same sample was measured repeatedly
☐	☒	The statistical test(s) used AND whether they are one- or two-sided<br>*Only common tests should be described solely by name; describe more complex techniques in the Methods section.*
☒	☐	A description of all covariates tested
☒	☐	A description of any assumptions or corrections, such as tests of normality and adjustment for multiple comparisons
☐	☒	A full description of the statistical parameters including central tendency (e.g. means) or other basic estimates (e.g. regression coefficient) AND variation (e.g. standard deviation) or associated estimates of uncertainty (e.g. confidence intervals)
☐	☒	For null hypothesis testing, the test statistic (e.g. *F*, *t*, *r*) with confidence intervals, effect sizes, degrees of freedom and *P* value noted<br>*Give P values as exact values whenever suitable.*
☒	☐	For Bayesian analysis, information on the choice of priors and Markov chain Monte Carlo settings
☒	☐	For hierarchical and complex designs, identification of the appropriate level for tests and full reporting of outcomes
☒	☐	Estimates of effect sizes (e.g. Cohen's *d*, Pearson's *r*), indicating how they were calculated

Our web collection on statistics for biologists contains articles on many of the points above.

Software and code

Policy information about availability of computer code

Data collection	We used instrument software to collect most experiments. Shimadzu Labsolutions were used for GC and HPLC data collection. Fluorescence data were collected using the Tecan 200 plate reader software 3.9.1.0.
Data analysis	We used Microsoft Excel 2016 and Origin2020 for data averaging, calculation of standard deviations, and evaluation of statistical significance.

For manuscripts utilizing custom algorithms or software that are central to the research but not yet described in published literature, software must be made available to editors and reviewers. We strongly encourage code deposition in a community repository (e.g. GitHub). See the Nature Portfolio guidelines for submitting code & software for further information.

Data

Policy information about availability of data

All manuscripts must include a data availability statement. This statement should provide the following information, where applicable:

- Accession codes, unique identifiers, or web links for publicly available datasets
- A description of any restrictions on data availability
- For clinical datasets or third party data, please ensure that the statement adheres to our policy

The authors declare that all data supporting the findings of this study are available within the paper (and its Supplementary Information files). The source data underlying Figs. 2-6, Supplementary Figs. 3-7 are provided in the Source Data File. All strains and plasmids information are deposited in Supplementary Data 1. The additional reactions and metabolites used in Oleo_iML1515 than iML1515 are deposited in Supplementary Data 2 and 3. All plasmid maps including annotations of oligonucleotide and gene sequences are deposited in the Supplementary Data 4.

Field-specific reporting

Please select the one below that is the best fit for your research. If you are not sure, read the appropriate sections before making your selection.

- [x] Life sciences
- [ ] Behavioural & social sciences
- [ ] Ecological, evolutionary & environmental sciences

For a reference copy of the document with all sections, see nature.com/documents/nr-reporting-summary-flat.pdf

Life sciences study design

All studies must disclose on these points even when the disclosure is negative.

Sample size	No sample size calculation was determined. Sample sizes in each round of experiment were limited by the number of sequences and by resources such as incubator space. Typically samples were tested in duplicate or triplicate, where cultures derived from separate, individual colonies.
Data exclusions	No data was excluded for analysis.
Replication	Data are presented as mean value and standard deviations of three independent biological replicates. The absorber coupling fed-batch fermentation experiments were performed twice. All attempts showed in this study were successful.
Randomization	For in vivo experiments, 3 individual colonies were picked randomly from the agar plates. In collection of GC and HPLC data the order of the samples was randomized.
Blinding	Blinding does not apply to our experiments for several reasons. We rationally designed the experiments to achieve our goal. we need to know the performance of each engineered strains. The samples of bacterial cultures were random sampling. The experiments did not involve any animals or human participants and the analysis was carried out entirely on bacteria.

Reporting for specific materials, systems and methods

We require information from authors about some types of materials, experimental systems and methods used in many studies. Here, indicate whether each material, system or method listed is relevant to your study. If you are not sure if a list item applies to your research, read the appropriate section before selecting a response.

Materials & experimental systems

n/a	Involved in the study	
[x]	[ ]	Antibodies
[x]	[ ]	Eukaryotic cell lines
[x]	[ ]	Palaeontology and archaeology
[x]	[ ]	Animals and other organisms
[x]	[ ]	Human research participants
[x]	[ ]	Clinical data
[x]	[ ]	Dual use research of concern

Methods

n/a	Involved in the study	
[x]	[ ]	ChIP-seq
[x]	[ ]	Flow cytometry
[x]	[ ]	MRI-based neuroimaging
